# Platelets activation is associated with elevated plasma mitochondrial DNA during cardiopulmonary bypass

**DOI:** 10.1186/s13019-016-0481-4

**Published:** 2016-06-07

**Authors:** Chaoyi Qin, Jun Gu, Jia Hu, Hong Qian, Xu Fei, Yajiao Li, Ruiqi Liu, Wei Meng

**Affiliations:** Department of Cardiovascular Surgery, West China Hospital, Si Chuan University, Guo Xue Alley 37, Cheng du, Sichuan 610041 People’s Republic of China; Department of Anesthesiology, Chengdu Women and Children’s Central Hospital, Chengdu, People’s Republic of China; Department of Cardiology, West China Hospital, Sichuan University, Chengdu, 610041 People’s Republic of China; Department of Burns and Plastic Surgery, West China Hospital, Sichuan University, Chengdu, 610041 People’s Republic of China

**Keywords:** Platelets, Mitochondrial DNA, Inflammation, Cardiopulmonary bypass

## Abstract

**Background:**

Mitochondrial DNA (mtDNA) was reported as a pro-inflammatory agent. In our previous study, elevation of plasma mtDNA was revealed after cardiac surgery with cardiopulmonary bypass (CPB). Platelets were activated during the cardiac surgery and recent study revealed its ability to release mtDNA. Our present study postulated that the elevated plasma mtDNA comes from activated platelets, which plays a critical role in post-CPB inflammatory responses.

**Methods:**

Sixty-eight patients who underwent coronary artery bypass graft (CABG) with CPB were enrolled in our study. Blood samples were collected before induction of anaesthesia (T1), at the end of CPB (T2), 12 h post-CPB (T3), 24 h post-CPB (T4), 48 h post-CPB (T5) and 72 h post-CPB (T6). Blood samples were analyzed for the routine blood test and prepared for plasma isolation. MtDNA concentration was measured by rt-PCR, and TNF-α and IL-6 were examined by specific ELISA kits. Subgroup study was analyzed by activation levels of platelet. Basic information, mtDNA level, TNF-α level and IL-6 level were all carefully studied in each quartile.

**Results:**

Activation level of platelets increased and peaked at T2, which decreased gradually from T3 to T6 (*P* < 0.05). MtDNA increased after CPB, peaked at T3, and then backed from T4 to T6 (*P* < 0.05). Bivariate correlation between peak activation level of platelets and peak plasma mtDNA level showed a positive correlation between these two parameters (*r* = 0.683, *P* < 0.0001). Both TNF-α and IL-6 showed similar patterns as mtDNA, with an increase from T1 to T3 and a decrease from T4 to T6 (*P* < 0.05). Subgroup analysis further demonstrated that patients with higher activation levels of PLT had higher plasma mtDNA levels and inflammatory level (*P* < 0.05).

**Conclusions:**

Our study revealed the dynamic changes of activation level of platelets and identified the interesting association between platelets activation and plasma mtDNA, suggesting a novel potential mechanism of activated platelets-induced post-CPB inflammatory responses.

## Background

Cardiac surgery under cardiopulmonary bypass (CPB) was firstly practiced in 1955 and reported by Kirklin JW, providing a new horizon for modern cardio-thoracic surgeries [1]. CPB can help bypass the circulation out of the heart and the lung, which offers a clear vision and stable condition for operation [2]. Although the low mortality for CPB is already analyzed, many harmful events were still reported. Myocardial ischemic/reperfusion injury (IRI) and abnormal status of circulation are two major causes for post-operative systemic inflammatory responses that take responsibilities for detrimental effects on the heart and distant organs [3–5]. Much endeavor has already been taken to decrease these negative effects. However, there is still lack of a sufficient way to control post-operative systemic inflammatory responses.

Mitochondria are considered to be organelles evolved both in saprophytic bacteria and endosymbiosis [6]. Mitochondrial DNA (mtDNA) consists of 16569 nucleotide bases which code 13 polypeptides [7]. Containing of CpG DNA repeats, mtDNA is widely studied as a pro-inflammatory agent. Many experiments report elevated plasma mtDNA after injuries, which is released into the circulation causing systemic inflammation [8, 9]. It is demonstrated that plasma mtDNA level can be used to improve risk prediction and predict mortality [10].

Activation of platelets is revealed during the cardiac surgery with CPB [11]. Dysfunction of platelets and severely decreased platelets are also reported after CPB [12]. Many studies are reported to use mean platelet volume/platelet count (MPV/platelet count) to represent the activation level of platelets [13]. It is demonstrated that activated platelets could release many pro-inflammatory cytokines causing systemic inflammatory responses [14]. Recently, Luc H. Boudreau, et al informed that activated platelets could release mtDNA into the circulation and promote inflammation through serving as substrates for bactericidal group IIA [15].

Our previous study has revealed the variation of plasma mtDNA level during and after cardiac surgery with CPB. In the present study, given the mtDNA releasing property of activated platelets, we postulated that activated platelets might, at least partially, take the responsibility for the elevated plasma mtDNA during the cardiac surgery with CPB. The present study aimed to explore whether platelets activation was associated with changes in levels of plasma mtDNA during CPB. Meanwhile, we also examined the relationship between platelet activation and inflammatory cytokines during CPB.

## Method

### Study population

Sixty-eight patients requiring coronary artery bypass graft (CABG) with CPB were included consecutively in our study from January 2014 to January 2015, who were admitted to the Department of Cardiovascular Surgery, West China Hospital, Sichuan University. Medical history including endocarditis, diabetes, hypertension, any infectious diseases, chronic inflammatory diseases, hematologic disorders that affect platelet count or platelets function, autoimmune diseases, post-operative acute renal failure, low cardiac output syndrome were designed as the excluding criteria. Pre-operative laboratory tests and examinations were carefully arranged and all patients had normal functions of kidney, liver and lung. Informed consents were signed by every patient or their family members. Post-operative standard care in cardiac intensive unit (CICU) were scheduled as the routine element for all patients. This protocol was conducted following the Declaration of Helsinki and approved by the West China Hospital Ethics Committee (*No.* 2012 (150)).

### Collection of blood samples

Blood samples were collected in two EDTA-coated blood collection tubes before induction of anaesthesia (T1), at the end of CPB (T2), 12 h post-CPB (T3), 24 h post-CPB (T4), 48 h post-CPB (T5) and 72 h post-CPB (T6). One blood collection tube was for the routine blood test immediately and results were reported by the Division of Clinical Hematology, West China Hospital, Sichuan University. The other tube was also immediately centrifuged at 1000 rpm/min for 15 min at 4 °C and the supernatant was transferred to a new tube as plasma without touching the pellet or the bottom of the tube. All plasma samples were rapidly stored in −80 °C freezer (Thermo, USA) and ready for the DNA isolation and ELISA assay. All procedures were conducted carefully to avoid contamination.

### DNA isolation and rt-PCR assay

The whole plasma DNA was isolated from plasma using the DNeasy Blood and Tissue Kit (#69504, Qiagen) as our previous report [16]. Briefly, 50 μL plasma sample was added to 50 μL phosphate buffered saline (PBS) and then centrifuged at 16000 g for 15 min at 4 °C. 90 μL of supernatant were kept for the next procedures. The rest procedures were performed exactly according to the manufacture’s protocol. At the last step, 200 μL elution buffer was added to resolve the DNA.

Plasma mtDNA levels were measured by SYBR-green dye-based rt-PCR assay using a PRISM 7300 sequence detection system. The primer sequences detecting mtDNA were human NADH dehydrogenase 1 gene: forward CGAGCAGTAGCCCAAACAAT, reverse TGTGATAAGGGTGGAGAGGTT. Plasmid DNA with complementary DNA sequence for human mtDNA was obtained from ORIGENE (SC101172, USA). Concentration of plasma mtDNA were converted to copy number via a DNA copy number calculator (http://cels.uri.edu/gsc/cndna.html; University of Rhode Island Genomics and Sequencing Center). Plasmid DNA was diluted in 10-fold serial dilutions and used as the standard curve. Every samples were studied three times for quality control.

All samples were measured with standards at the same time. Plasma mtDNA levels were shown in copies per microliter of plasma (copies/μL) according to the following formula:

$$ \mathrm{c} = \mathrm{Q}\ *\ {\mathrm{V}}_{\mathrm{DNA}}/{\mathrm{V}}_{\mathrm{PCR}}*\ 1/{\mathrm{V}}_{\mathrm{ext}} $$

where c is the concentration of plasma mtDNA (copies/μL); Q means quantity of DNA measured by rt-PCR; V_DNA_ means the total volume of plasma DNA solution obtained from extraction, 200 μL in this study; V_PCR_ means the volume of plasma DNA solution for rt-PCR, 1 μL in this study; V_ext_ means the volume of plasma used for extraction, 50 μL in this study.

### Plasma TNF-α and IL-6 measurement

Plasma TNF-α and IL-6 was measured by the enzyme-linked immunosorbent assay (ELISA) kits, specifically designed for human TNF-α and IL-6, respectively (Solarbio, China). All procedures were carried out following the standard protocols (included in ELISA kits). Three duplicated samples were set and all samples were measured with serial diluted standards at the same time. Spectrophotometry (VARIOSKAN, Thermo, USA) was used to detect the intensity of the transmitted light. Data was expressed in picogram per mL (pg/mL).

### Statistical analysis

All descriptive data was shown as mean ± standard error of the mean (SEM) and analyzed with SPSS statistical software version 20.0 for Windows (SPSS, Inc. Chicago, IL, USA). All descriptive data was near-normal by SPSS analysis. Multiple comparisons were analyzed by the one-way ANOVA followed by Bonferroni’s test. The Pearson correlation coefficient test was performed to analyze the relationship between MPV/platelet count and plasma mtDNA. Data (platelet count, mtDNA, TNF-α, IL-6) collected after T1 was corrected by hematocrit (Hct) at T1 due to the hemodilution during cardiac surgery (Data_corrected_ = Data_collection_ × Hct_T1_/Hct_Tx_). *P* < 0.05 was considered as statistically significant.

## Result

### Baseline information

Basic characteristics, surgical information and laboratory results of all patients were presented in Table 1. All sixty-eight patients were successfully performed CABG with CPB. No patient died or needed a second surgery during the hospitalization. The average age and BMI were (47.5 ± 15.2) years old and (24.1 ± 3.5). During surgery, the average time of aortic cross-clamping and total CPB were (64.3 ± 21.7) min and (96 ± 29.1) min, respectively. On the first day admission, basic laboratory data such as serum creatinine, total cholesterol (TC), high density lipoprotein (HDL), low density lipoprotein (LDL), triglycerides (TG), glucose, red blood cell count (RBC), white blood cell count (WBC), platelet count, MPV, hemoglobin (Hb) and Hct was collected and represented.

**Table 1:** Baseline information

Characteristics	*N* = 68
Age (years)	47.5 ± 15.2
Women	41
BMI	24.1 ± 3.5
BSA (m^2^)	1.8 ± 0.2
Stable angina	12
Unstable angina	18
STEMI	24
NSTEMI	14
Surgical information	
Aortic cross-clamping time (min)	64.3 ± 21.7
Total CPB time (min)	96.0 ± 29.1
Laboratory data	
serum creatinine (μmol/L)	0.6 ± 0.3
Total cholesterol (mg/dL)	200.7 ± 41.3
HDL (mmol/L)	1.1 ± 0.3
LDL (mmol/L)	3.0 ± 0.5
TG (mmol/L)	1.7 ± 0.3
Glucose (mg/dL)	138.1 ± 31.4
RBC (×10^12^/L)	4.3 ± 0.4
WBC (×10^12^/L)	5.8 ± 1.3
platelet count (×10^9^/L)	203 ± 49
MPV (fL)	10.2 ± 1.1
Hemoglobin (g/dL)	12.7 ± 1.4
Hematocrit (%)	38.3 ± 4.7

Variables are presented as mean ± SD or as numbers. *BMI* body mass index, *BSA* body surface area, *STEMI* ST-segment myocardial infarction, *NSTEMI* Non-ST-segment myocardial infarction, *CPB* cardiopulmonary bypass, *HDL* high density lipoprotein, *LDL* low density lipoprotein, *TG* triglycerides, *RBC* red blood cell, *WBC* white blood cell, *MPV* mean platelet volume

### Dynamic monitoring the activation of platelets

**Fig. 1:**
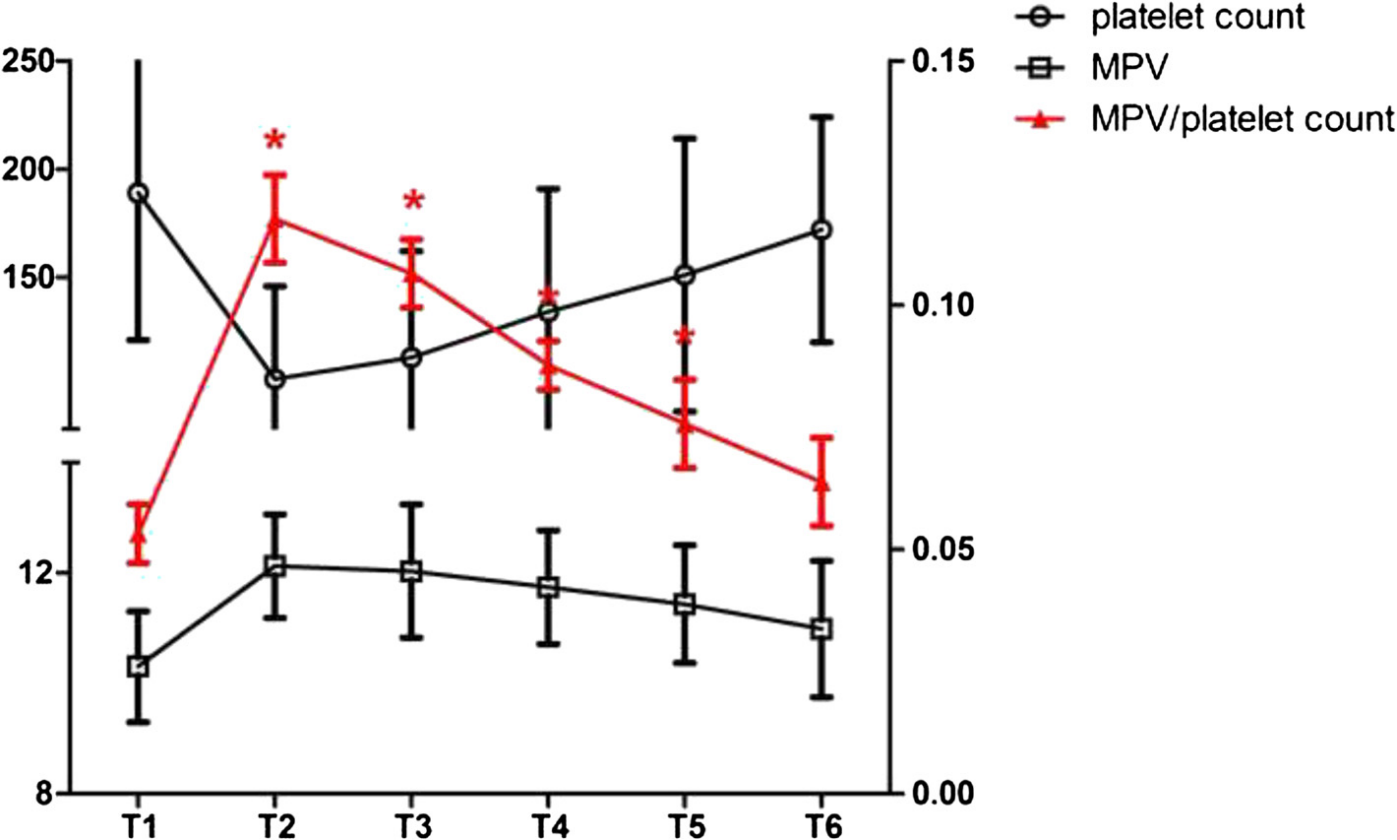
Changes of perioperative platelets activation level. Platelets count decreased significantly at the end of CPB (T2) and increased from T3 to T6 (*P* < 0.05). Mean platelet volume (MPV) increased at T2 and then back to normal gradually (*P* < 0.05). MPV/platelet count increased significantly at T2 and decreased from T3 to T6 (*P* < 0.05). *P < 0.05 vs. T1. T1: before induction of anaesthesia; T2: at the end of CPB; T3: 12 h post-CPB; T4: 24 h post-CPB; T5: 48 h post-CPB; T6: 72 h post-CPB. Units of left y axis (platelet count): ×10^9^ per litre; Units of left y axis (MPV): femtoliter (fl)

MPV/platelet count ratio was used to represent the activation level of platelets during the cardiac surgery. Through serial six blood samples for each patient, dynamic changes of activation level of platelets were monitored during the cardiac surgery with CPB. As shown in Fig. 1, platelet count, MPV and MPV/platelet count ratio were presented. Compared with T1, platelet count significantly decreased and touched the bottom at T2 (*P* < 0.05) following a gradually increasing back to the baseline level from T3 to T6. On the contrary, MPV increased significantly and reached the highest level at T2 comparing with T1 (*P* < 0.05), which dropped back to normal gradually. Therefore, MPV/platelet count climbed to the peak at T2 and decreased gradually (*P* < 0.05). Our data demonstrated that platelets were activated during the cardiac surgery with CPB and the highest activation level of platelets occurred at the end of CPB.

### Plasma mtDNA changes and its correlation with peak MPV/platelet count

**Fig. 2:**
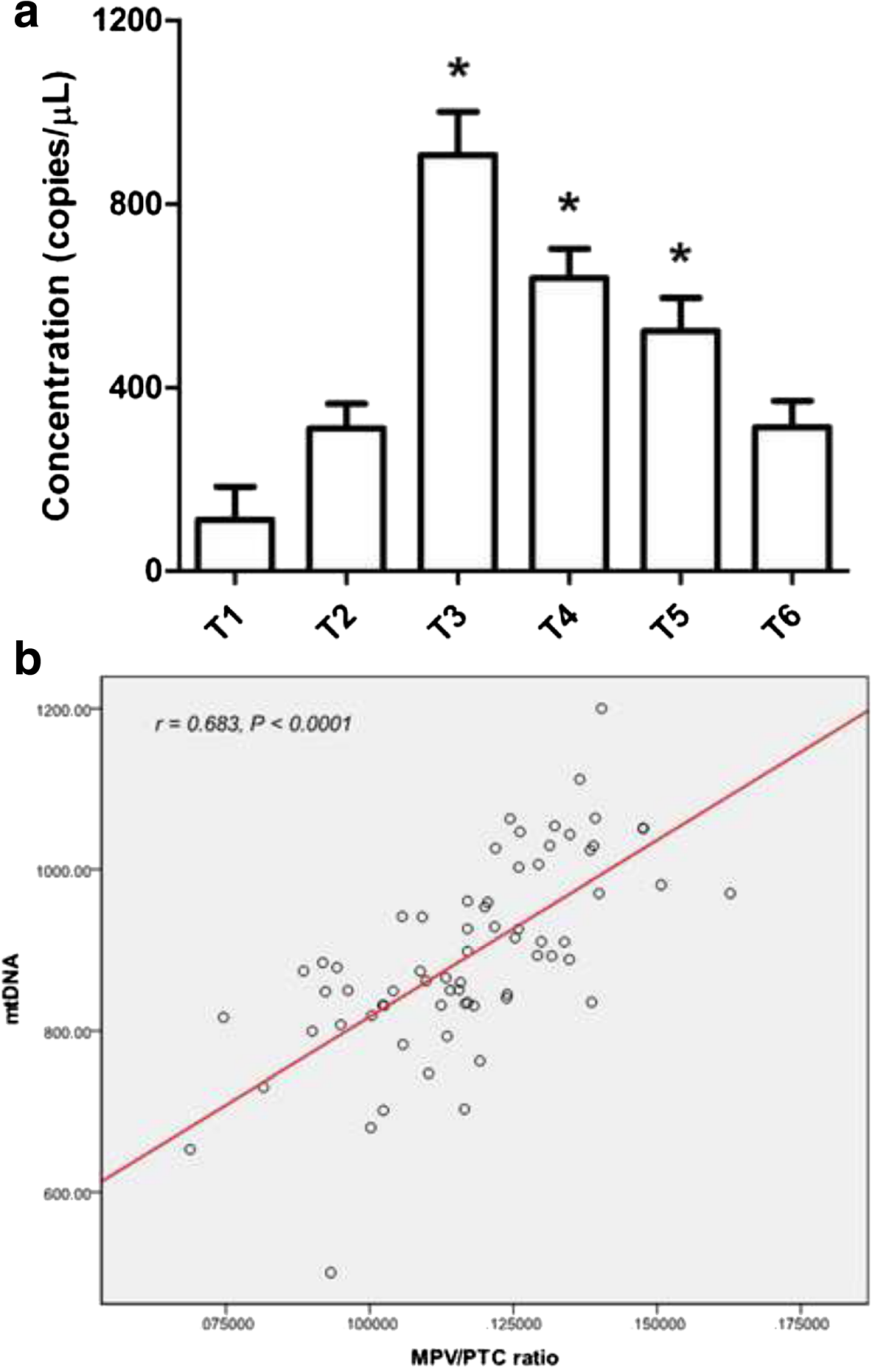
**a** Variation of plasma mtDNA during the cardiac surgery with CPB and the correlation between peak activation level of platelets and peak plasma mtDNA level. Plasma mtDNA level increased significantly and peaked at T3, which decreased slowly from T3 to T6 (*P* < 0.05). **b** Bivariate correlation study showed a positive correlation between peak activation level of platelets and peak plasma mtDNA level (*r* = 0.683, *P* < 0.0001). *P < 0.05 vs. T1. Units of y axis: copies per microliter (copies/μL)

Plasma samples were used to examine plasma mtDNA levels by using rt-PCR technique (Data was shown in Fig. 2a). As presented, changes of plasma mtDNA was similar to MPV/platelet count, which increased significantly after T1 and decreased gradually from T4 to T6 (*P* < 0.05). However, the highest point happened at T3 for plasma mtDNA.

To illustrate the relationship between platelets activation and plasma mtDNA, bivariate correlation analysis was used. Since we excluded other possible complications that might disturb our results, the peak MPV/platelet count and peak plasma mtDNA were used to stand for platelets activation level and mtDNA releasing level, respectively, during the cardiac surgery with CPB. As shown in Fig. 2b, positive correlation was analyzed between these two parameters (*r* = 0.683, *P* < 0.001). These data demonstrated a close relationship between platelets activation and plasma mtDNA.

### Levels of TNF-α and IL-6

**Fig. 3:**
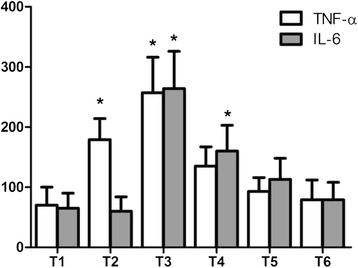
Merged curve of perioperative inflammatory cytokines in the cardiac surgery with CPB. Both TNF-α and IL-6 were elevated significantly after CPB (*P* < 0.05). Levels of TNF-α and IL-6 peaked at T3 (*P* < 0.05). *P < 0.05 vs. respective T1. Units of y axis: picogram per milliliter (pg/mL)

Plasma TNF-α and IL-6 levels were measured by ELISA kits in all plasma samples. Data was presented in Fig. 3. Both TNF-α and IL-6 increased since T2 and got the highest level at T3, followed by a gradual decreasing from T4 to T6 (*P* < 0.05). Consistence with previous studies, our study confirmed dynamic changes of perioperative plasma TNF-α and IL-6 levels.

### Subgroup analysis

**Fig. 4:**
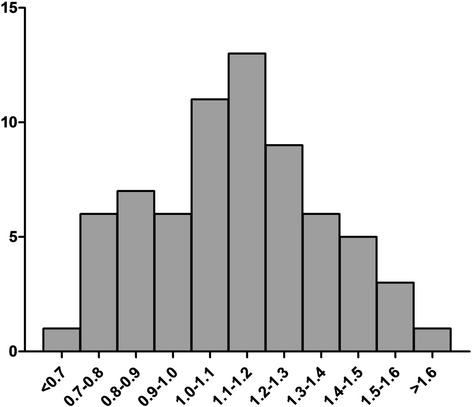
Distribution of study population according to the activation level of platelets

According to our study, activation level of platelets peaked at the end of CPB (T2) and the mean value of MPV/platelet count was 0.12 ± 0.019 (0.069–0.162). Distribution of study population was shown in Fig. 4. Subgroups were defined as follow: Quartile 1, MPV/platelet count <0.09 (*N* = 14); Quartile 2, MPV/platelet count = 0.09–0.11 (*N* = 17); Quartile 3, MPV/platelet count = 0.11–0.13 (*N* = 22); Quartile 4, MPV/platelet count >0.13 (*N* = 15).

We analyzed the baseline information for each quartile, shown in Table 2. Average age, BMI, time of aortic cross-clamping, total CPB, serum creatinine, TC, HDL, LDL, TG, glucose, RBC, WBC, Hb and Hct were carefully calculated and showed no significance between four quartiles. Meanwhile, plasma mtDNA level, TNF-α and IL-6 at 12 h post-CPB (T3) were also included to compare among four quartiles. Intriguingly, significant differences were observed among four quartiles. In general, patients in the higher MPV/platelet count quartiles had higher level of plasma mtDNA, TNF-α and IL-6 (*P* < 0.05). The present study indicated that higher levels of plasma mtDNA, TNF-α and IL-6 were related to the higher MPV/platelet count quartiles, further implying that mtDNA might come from activated PLT and play a pro-inflammatory role.

**Table 2:** Subgroup analysis

	MPV/platelet count			
	Quartile 1	Quartile 2	Quartile 3	Quartile 4
	< 0.09	0.09–0.11	0.11–0.13	> 0.13
Number	14	17	22	15
Age (years)	46.5 ± 9.2	47.1 ± 6.0	47.8 ± 8.3	48.4 ± 12.5
Women	9	9	15	8
BMI	23.4 ± 3.5	24.1 ± 2.7	24.5 ± 3.1	23.7 ± 3.8
BSA (m^2^)	1.7 ± 0.2	1.8 ± 0.3	1.8 ± 0.2	1.8 ± 0.3
Stable angina	3	3	4	2
Unstable angina	4	5	5	4
STEMI	5	5	8	6
NSTEMI	2	4	5	3
Surgical information				
Aortic cross-clamping time (min)	64.3 ± 21.7	63.1 ± 20.6	64.7 ± 22.3	66.3 ± 19.8
Total CPB time (min)	96.0 ± 26.3	96.4 ± 28.1	96.1 ± 25.9	98.3 ± 30.2
Laboratory data				
serum creatinine (μmol/L)	0.6 ± 0.2	0.6 ± 0.3	0.5 ± 0.3	0.6 ± 0.2
TC (mg/dL)	203.7 ± 31.6	200.1 ± 39.3	201.4 ± 36.7	207.2 ± 45.3
HDL (mmol/L)	1.2 ± 0.4	1.2 ± 0.2	1.3 ± 0.3	1.3 ± 0.2
LDL (mmol/L)	3.1 ± 0.5	2.9 ± 0.4	3.0 ± 0.4	3.0 ± 0.4
TG (mmol/L)	1.8 ± 0.3	1.7 ± 0.4	1.7 ± 0.2	1.8 ± 0.3
Glucose (mg/dL)	132.1 ± 31.4	139 ± 30.7	121.5 ± 36.3	145.3 ± 31.1
RBC (×10^12^/L)	4.4 ± 0.4	4.2 ± 0.3	4.3 ± 0.3	4.1 ± 0.5
WBC (×10^12^/L)	5.6 ± 1.5	4.9 ± 1.7	6.4 ± 1.3	6.0 ± 1.0
Hemoglobin (g/L)	125.2 ± 11.9	123.8 ± 14.9	130.1 ± 11.3	122.5 ± 10.1
Hematocrit (%)	38.2 ± 3.7	38.1 ± 3.2	38.4 ± 5.3	37.2 ± 3.9
MPV/platelet count ratio	0.08 ± 0.009	0.10 ± 0.010	0.12 ± 0.009	0.14 ± 0.011
Plasma mtDNA (copies/μL)	798 ± 49	889 ± 65^*^	953 ± 87^*^	1021 ± 58^*^
TNF-α (pg/mL)	164 ± 39	204 ± 42^*^	294 ± 37^*^	337 ± 29^*^
IL-6 (pg/mL)	181 ± 43	223 ± 38^*^	298 ± 42^*^	328 ± 37^*^

Variables are presented as mean ± SD or as numbers. *BMI* body mass index, *BSA* body surface area, *STEMI* ST-segment myocardial infarction, *NSTEMI* Non-ST-segment myocardial infarction, *CPB* cardiopulmonary bypass, *TC* total cholesterol, *HDL* high density lipoprotein, *LDL* low density lipoprotein, *TG* triglycerides, *RBC* red blood cell, *WBC* white blood cell, *MPV* mean platelet volume, *TNF-α* tumor necrosis factor-α, *IL-6* interleukin-6. ^*^ *P* < 0.05 vs. respective Quartile 1

## Discussion

In our present study, we first dynamically monitored the activation level of platelets by analyzing MPV/platelet count ratio during the cardiac surgery with CPB, showing that platelets activation level peaked at the end of CPB. In addition, like our previous study, we revealed the variation of plasma mtDNA and inflammatory cytokines (TNF-α and IL-6). To study the possible source of plasma mtDNA, bivariate correlation analysis was used and demonstrated that the peak MPV/platelet count ratio was positively correlated with the peak plasma mtDNA level. Subgroup analysis also suggested that patients with CPB with higher peak MPV/platelet count ratio had higher post-CPB plasma mtDNA level and higher systemic inflammatory level.

During cardiac surgery with CPB, the blood flows out of the vessels and goes through the circuits. A large number of studies demonstrated that exposure of blood to the bypass circuit surface initiated systemic inflammatory responses, taking responsibility for post-CPB inflammation and organ dysfunction [17]. It is well known that the activation of platelets during the CPB could cause post-CPB inflammatory responses. Additionally, some studies revealed that activated platelets played its role in inflammation via expression of P-selectin and CD40 ligand [18, 19]. Recently, Procter NE, et al identified that the hyper-aggregability of platelets could cause inflammation through MPO-related pathway in patients with atrial fibrillation [20]. Therefore, anti-coagulation strategy was widely used in the cardiac surgery with CPB and heparin-coated circuits were also applied to abate the inflammation and coagulation [21]. In our study, we illustrated the dynamic changes of platelets activation during the cardiac surgery with CPB. Unsurprisingly, rapid activation of platelets was shown and activation level peaked at the end of CPB. Meanwhile, post-CPB inflammatory level was also shown in our study and the peak level of inflammatory responses occurred at 12 h post-CPB, which is 12 h later than the peak point of platelets activation. Given the well-known pro-inflammatory properties of activated platelets, our data suggested that activation of platelets might promote post-CPB inflammatory responses.

Although many studies focused on the mechanism between activated platelets and inflammation, it was still not fully understood. In our previous study, a tight and strong relationship between post-CPB plasma mtDNA level and plasma inflammatory cytokines was revealed. Therefore, elevated plasma mtDNA was regarded as a pro-inflammatory agent during the cardiac surgery with CPB and promoted post-CPB inflammatory responses. A study conducted by Luc H. Boudreau, et al suggested that activated platelets can release mitochondria and mtDNA. Transfused patients and in vitro platelets activation study were used to evaluate the mtDNA changes [15]. Platelets, as small discoid anucleate cell fragment from megakaryocytes, contained cytoplasmic organelles, including mitochondria. After the activation of platelets, intracellular components were released via the cytoplasmic granules formation and the fusion with the plasma membrane [22]. Given that the perioperative variation of plasma mtDNA level we studied, it is possible that most of post-CPB plasma mtDNA were from activated platelets. In our study, we found the peak platelets activation time is T2 and peak mtDNA level occurred at T3, which mean that the mtDNA elevation came after the platelets activation. Bivariate correlation analysis was applied to study the correlation between peak platelets activation level and peak plasma mtDNA level. Since the time gap and positive correlation, our results suggested that activated platelets might be the source of plasma mtDNA during the cardiac surgery with CPB.

MtDNA has been widely studied recently, which could cause systemic inflammation when it was released into the circulation [23]. Plasma mtDNA was captured into leukocytes and acts as bacteria to cause inflammatory responses [24]. Studies found that plasma mtDNA level can represent the invasiveness and complexation of surgeries [25]. In vivo study revealed that injection of mtDNA can cause severe systemic inflammatory responses and multiple organ dysfunction [23]. Consistent with our previous study, mtDNA level after cardiac surgery was correlated with inflammatory response level. And in the present study, we concluded that activated platelets may take responsibilities for the elevated mtDNA level. However, many other factors are also considered to account for elevated mtDNA. Many studies reported the myocardial ischemia/reperfusion injuries after cardiac surgery with CPB [26, 27]. During the ischemia/reperfusion injury, myocardial tissue damage may cause mtDNA releases. Also, the damage of surgery itself to the heart can cause tissue damage and mtDNA release.

In order to further study the relationship between activated platelets and plasma mtDNA, we conducted the subgroup analysis. Four quartiles according to platelets activation levels were set. Unsurprisingly, patients with higher platelets activation level presented higher level of plasma mtDNA and suffered higher level of post-CPB inflammation. Levels of TNF-α and IL-6 represented the severity of post-CPB inflammatory responses [28]. Post-CPB inflammation might cause a delay of the recovery and deteriorate the hospital outcomes [29]. Above all, our data revealed the tight association between platelets activation and elevated plasma mtDNA level, suggesting that activated platelets might cause post-CPB inflammation through releasing mtDNA.

Although we got some excellent results, our study still has many limitations. Our study only enrolled sixty-eight patients undergoing CABG with CPB. To further study the relationships among platelets, mtDNA and inflammatory responses, a large-scale study is needed. Additionally, more specific and statistical grouping and subgrouping setting should be designed. Moreover, we still need further experiments or clinic study to achieve the direct evidences to prove our speculation. Finally, our study can only provide a preliminary result about the role of mtDNA in platelets-mediated post-CPB inflammatory responses, but raise a clinical foundation for further relative studies.

## Conclusion

Our study demonstrated the positive correlation between platelets activation and peak plasma mtDNA level during the cardiac surgery with CPB, which explains the possible source of abundant plasma mtDNA after CPB. Therefore, in combination with our previous study, we raised a novel potential theory that mtDNA might act as a mediator between platelets and inflammation after cardiac surgery with CPB and platelets might act as a valuable target to modify the inflammation.
